# TotalSegmentator-AI for aortic segmentation in CT: Reliable performance in normal anatomy but limited utility in pathological aortic disease

**DOI:** 10.1016/j.ejro.2026.100772

**Published:** 2026-06-08

**Authors:** Dania El Rahal, Chiara Pozzessere, Marianna Gulizia, David C. Rotzinger, Guillaume Fahrni

**Affiliations:** Department of Diagnostic and Interventional Radiology, Lausanne University Hospital and University of Lausanne, Rue du Bugnon 46, Lausanne 1011, Switzerland

**Keywords:** Aorta, Artificial Intelligence, Deep Learning, Segmentation, CT Angiography, Clinical analysis

## Abstract

**Introduction:**

Accurate aortic segmentation on computed tomography angiography (CTA) is essential for diagnosing aortic disease, cardiovascular risk assessment, and surgical planning. Deep learning algorithms, such as TotalSegmentator-AI, offer fully automated multi-organ segmentation, yet their performance in pathological aortic conditions remains uncertain. This study performs a clinical stress-test of TotalSegmentator-AI, mapping its boundaries and structural failure modes across a spectrum of normal and pathological cases.

**Methods:**

In this monocentric, retrospective study, 60 CTA scans from 2014 to 2024 were categorized into six groups: young, elderly, aneurysm, dissection, venous phase, and non-contrast phase. TotalSegmentator-AI was applied without manual correction. Two radiologists independently rated six aortic segments per scan using a five-point qualitative scale. Quantitative segmentation errors were correlated with qualitative scores using Spearman’s correlation, and inter-reader agreement was assessed with weighted Cohen’s kappa.

**Results:**

All scans were successfully processed, yielding 360 aortic segments. Median segmentation quality was 4 [IQR 4–5], with 77% rated good or excellent. Performance was consistent across segments (p = 0.16) but varied by category (p < 0.001): best in young patients (5 [IQR 4–5]) and adequate in non-contrast and venous-phase scans (4 [IQR 4–5]), poorest in dissections (3 [IQR 3–4]) and aneurysms (4 [IQR 3–4]). A strong negative correlation was observed between qualitative scores and quantitative errors (ρ = –1, p = 0.017). Inter-reader agreement was substantial (κ = 0.72).

**Conclusion:**

TotalSegmentator-AI achieves accurate aortic segmentation in normal anatomy but is inadequate for unsupervised clinical use in complex pathologies like aneurysms and dissections. Comprehensive human-in-the-loop quality control or dedicated pathology-inclusive models are mandatory before AI-based segmentation can be safely integrated into vascular clinical workflows.

## 1 Introduction

Automated anatomical segmentation in computed tomography (CT) images is a fundamental process for patient diagnosis, surgical planning, and treatment monitoring, traditional medical imaging workflows being increasingly enhanced by deep learning and convolutional neural networks [1], [2], [3]. Long performed manually or semi-automatically, segmentation now relies on these automated algorithms to detect and isolate complex structures with remarkable accuracy. This transition addresses several critical clinical challenges: improving diagnostic precision, enhancing the reproducibility of analyses, and reducing the cognitive burden on radiologists facing escalating workloads [4].

Among the organs that pose particular segmentation challenges is the aorta, a major blood vessel whose anatomy and appearance on imaging can vary significantly across individuals, pathological conditions (such as aneurysms, dissections, atherosclerosis), age groups, and contrast injection phases [5], [6]. Yet, accurate aortic segmentation is crucial in numerous clinical contexts, particularly for vascular risk assessment and endovascular intervention planning [7].

In this context, innovative tools such as TotalSegmentator-AI have emerged [8], [9]. Based on convolutional neural networks (CNNs) trained on large-scale public datasets like those from the Medical Segmentation Decathlon [10], this tool aims to provide fast, generalizable, and fully automated multi-organ segmentation. Initial evaluations suggest promising results in standard cases. However, while promising, its performance in complex pathological conditions, elderly or comorbid populations, or medium-quality images remains insufficiently explored [11].

The clinical transferability of these AI models, often trained on "idealized" datasets, thus represents a key research priority. Understanding their limitations, particularly in segmenting sensitive structures like the aorta, is essential to ensuring their safe and ethical integration into routine medical practice [12].

Thus, the objective of this study was to perform a clinical "stress-test" to systematically map the boundaries and failure modes of TotalSegmentator-AI for aortic segmentation in routine clinical CT scans. We hypothesized that while the tool might perform adequately in standardized, non-pathological anatomy, its reliability would break down in complex, real-world pathological conditions (e.g., aneurysms, dissections) where accurate segmentation is most critically required for clinical decision-making.

## 2 Materials and methods

### 2.1 Study design and cases selection

**Fig. 1:**
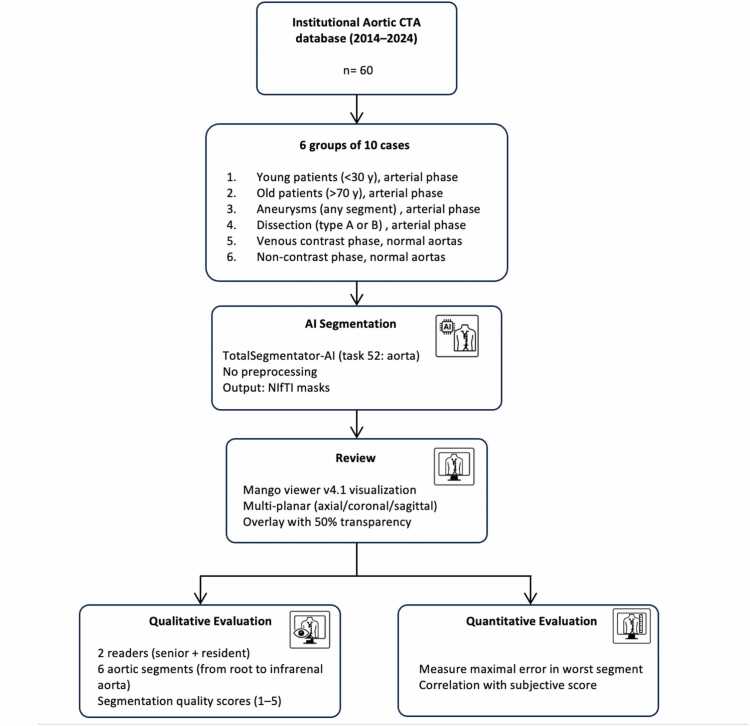
Study Flowchart.

This monocentric, retrospective study was conducted at a tertiary care academic hospital and was approved by the local ethics committee (protocol ID: AORTASEG−23–01–2025). A total of 60 thoraco-abdominal computed tomography angiography (CTA) scans were retrospectively selected from the institutional radiology archive, covering a ten-year period from 2014 to 2024. The dataset was manually selected to reflect a broad spectrum of aortic morphologies, including both normal and pathological configurations. Pathological configurations were heterogeneous, including aneurysms of varying extent and dissections with complex true–false lumen anatomy, reflecting real-world segmentation challenges. Cases were identified by screening the institutional radiology database using predefined keywords in radiology reports (e.g., “aortic aneurysm”, “aortic dissection”, “thoracoabdominal aneurysm”). For pathological cases, the presence of aortic disease was subsequently confirmed by visual review of the CT images. For non-pathological cases, reports were screened for the absence of aortic pathology, and normal aortic anatomy was verified on imaging. The cases were grouped into six categories of ten patients each: 1) young individuals under 30 years of age with normal aortic morphology in arterial phase imaging; 2) elderly patients over 70 years with age-appropriate aortic changes considered within normal limits, also imaged in the arterial phase; 3) patients with aortic aneurysms involving one or more aortic segments; 4) patients with untreated Stanford type A or B aortic dissections; 5) patients with normal aortas imaged during the venous phase; 6) patients with normal aortas assessed in non-contrast-enhanced CT scans. The dataset of 60 thoraco-abdominal aortic CTA scans used in this study is publicly available as an open-access dataset on the Zenodo platform [13]. The overall study design and patient selection process are illustrated in Fig. 1.

### 2.2 Imaging protocol and image segmentation

All included CTA scans were acquired using a 256-slice Revolution CT scanner (GE Healthcare, Chicago, USA) in helical mode with prospective ECG-gating. Acquisition parameters included a 230 mm small field of view (FOV) centered on the aorta, a 512 × 512 matrix, 1.25 mm slice thickness, a rotation time of 0.28 s, and tube voltages ranging from 80 to 120 kVp with automatic tube current modulation (Smart-mA, 200–900 mA). Image reconstruction was performed using a standard convolution kernel. For contrast-enhanced scans, intravenous iodinated contrast (Iohexol 350 mg I/mL) was administered according to institutional protocols (total volume of 80–100 mL depending on the patient’s body habitus and injected at a rate of 4 mL/s, arterial-phase acquisitions performed approximately 40 s after injection and venous phase acquisitions at approximately 90 s). Image segmentation was performed using the TotalSegmentator-AI algorithm, which applies deep learning-based semantic segmentation. The aortic segmentation task (task index = 52) was executed via a Python script on a local workstation without any pre-processing or manual adjustment of the data. The pipeline was run on a laptop workstation equipped with an Apple M1 Pro processor and 32 GB of unified memory, using the specialized sub-routing flag (--roi_subset aorta) to isolate the aortic volume. Under this setup, the average end-to-end processing and inference time was approximately 1 min per patient dataset, requiring no dedicated server infrastructure. The resulting segmentation masks were exported in compressed NIfTI format and co-registered with the original CT datasets, also in NIfTI format, for review. Segmentation overlays were visualized using the Multi-image Analysis GUI software (Mango v4.1, University of Texas Health Science Center at San Antonio), enabling multi-planar evaluation in axial, coronal, and sagittal views. The overlay was displayed with 80% transparency to allow simultaneous visualization of both the aortic lumen and the AI-generated contour, facilitating accurate assessment of segmentation errors.

### 2.3 Qualitative evaluation

**Fig. 2:**
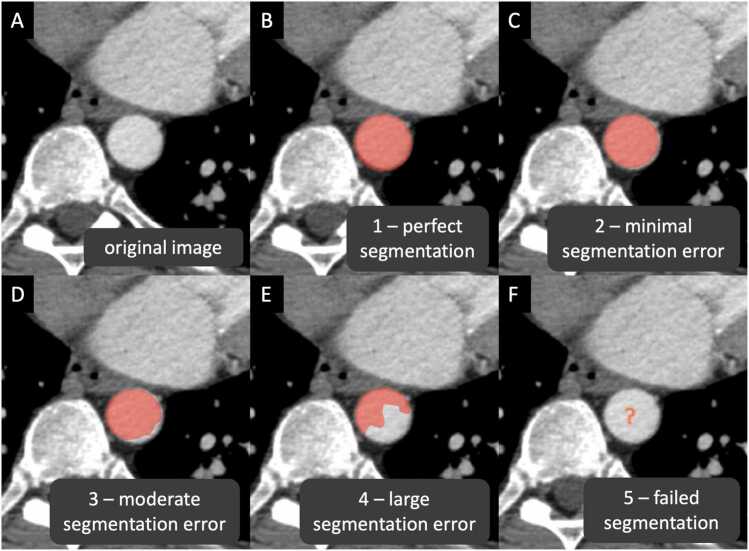
Schematic representation of the 5-point qualitative scoring system for AI-based aortic segmentation quality. (A) Baseline CTA image showing normal aorta prior to segmentation. (B-F) Segmentation masks (red transparent overlay) applied to the aortic lumen with progressive quality degradation: (B) Score 5: perfect segmentation with complete contour alignment, (C) Score 4: minimal gaps, (D) Score 3: moderate gaps, (E) Score 2: large gaps, (F) Score 1: failed segmentation.

The quality of the AI-generated aortic segmentations was independently assessed by two readers: one board-certified radiologist with eight years of experience in cardiovascular imaging (G.F) and one radiology resident with three years of experience (D.E.R). The two readers evaluated the datasets independently. While they were blinded to patient clinical records and exact chronological ages, formal blinding to the six imaging categories was not feasible due to the self-evident visual features of the underlying conditions (e.g., contrast phases or pathological structures) which are inherently recognizable to experienced radiologists." For each patient, the segmentation was evaluated across six predefined anatomical regions of the aorta: 1) the aortic root; 2) ascending aorta; 3) aortic arch; 4) descending thoracic aorta; 5) suprarenal abdominal aorta; 6) infrarenal abdominal aorta. Each region was rated on a five-point qualitative scale reflecting the degree of mismatch between the segmentation and the true aortic contour. A score of 5 denoted perfect contour alignment; 4 reflected minimal segmentation error (visually estimated at <2 mm); 3 indicated moderate segmentation error (approximately 2–5 mm); 2 represented large segmentation error (visually exceeding 5 mm); and 1 indicated complete segmentation failure of either the full segment or part of it. Segmentation error measurements were based on visual assessment rather than formal quantification. Representative examples for each score are illustrated in Fig. 2.

### 2.4 Quantitative evaluation

In parallel with the qualitative assessment, a quantitative analysis was conducted by one expert reader (G.F) to precisely measure the maximum discrepancy in millimeters between the AI-generated contour and the actual aortic wall. This measurement was performed on the single aortic segment that had received the lowest qualitative score for each case. The measured segmentation error values were then averaged across all cases within each qualitative score category. This allowed for a quantitative correlation between subjective assessment scores and the objective magnitude of segmentation error.

### 2.5 Statistical analysis

Statistical analysis was performed using R (v4.3.2, R Foundation for Statistical Computing, Vienna, Austria) through RStudio (v2024.09.0 +375). Scores variables were summarized using both median and interquartile range (IQR), as well as mean ± standard deviation (SD) to facilitate comparison. For comparisons of related variables across multiple anatomical segments, a non-parametric Friedman test was used. In order to evaluate the relationship between the quality of segmentation and the extent of the segmentation errors identified by the AI model on CT images of the aorta, a Spearman's rank correlation test was performed. Inter-observer agreement for the qualitative segmentation scores was assessed using the weighted Cohen’s kappa with squared weights. Kappa values were interpreted as follows: < 0 (no agreement), 0.01–0.20 (slight), 0.21–0.40 (fair), 0.41–0.60 (moderate), 0.61–0.80 (substantial), and 0.81–1.00 (almost perfect agreement). A p-value < 0.05 was considered statistically significant.

## 3 Results

**Fig. 3:**
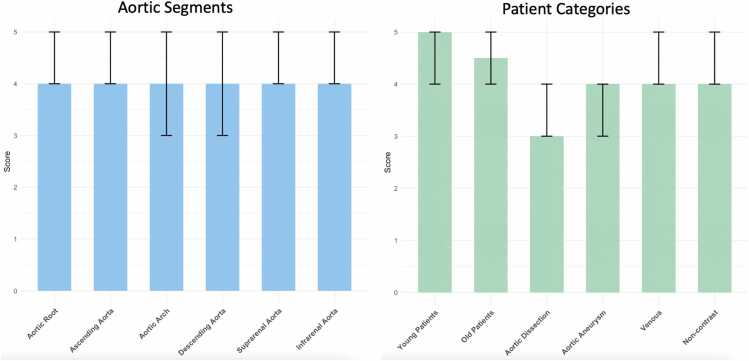
Comparison of image quality scores across aortic segments and patient groups. (Left) Scores for aortic segments, presented in anatomical order from proximal (aortic root) to distal (infrarenal aorta). (Right) Scores stratified by patient categories. Bars represent median scores, error bars show 25th−75th percentiles (IQR).

A total of 60 aortic CTA scans, covering 360 aortic segments, were evaluated independently by two readers. This resulted in 720 qualitative scores, reflecting assessments from both readers for each segment. Within the pathological cohorts, the aneurysm group consisted of 5 abdominal and 5 thoracoabdominal aneurysms, while the dissection group included 10 Stanford Type A dissections. All 60 aortic CTA scans were successfully processed by TotalSegmentator-AI without execution errors or technical failures. The distribution of scores across all segments was as follows: 252 (35%) received a grade of 5 (perfect alignment), 302 (42%) a grade of 4 (minimal error), 113 (16%) a grade of 3 (moderate error), 37 (5%) a grade of 2 (large error), and 16 (2%) a grade of 1 (segmentation failure). The overall median segmentation score across all aortas was 4 [IQR 4, 5], with a mean of 4.00 ± 0.96, indicating adequate performance. Segmentation results, separated in *per-segment* and *per-category* analysis, are available in Table 1 and Fig. 3.

**Table 1:** Segmentation quality scores by aortic segment and patient category.

	Median [IQR]	Mean (±SD)
Overall	4 [4,5]	4.00 (±0.96)
Per-Segment Analysis		
Aortic Root	4 [4,5]	4.09 (±0.79)
Ascending Aorta	4 [4,5]	4.04 (±0.97)
Aortic Arch	4 [3,5]	3.95 (±1.01)
Descending Aorta	4 [3,5]	3.90 (±0.98)
Suprarenal Aorta	4 [4,5]	4.11 (±0.91)
Infrarenal Aorta	4 [4,5]	4.05 (±1.04)
Per-Category Analysis		
Young Patients	5 [4,5]	4.58 (±0.53)
Elderly Patients	4.5 [4,5]	4.33 (±0.81)
Aortic Aneurysm	4 [3,4]	3.61 (±0.94)
Aortic Dissection	3 [3,4]	3.22 (±1.08)
Venous Phase	4 [4,5]	4.18 (±0.74)
Non-Contrast	4 [4,5]	4.23 (±0.83)

### 3.1 Per-segment analysis

Segmentation quality varied slightly across aortic segments, with no notable differences. The median score was consistently 4 for all segments, with interquartile ranges (IQR) spanning 3–5. Specifically, Aortic Root and Ascending Aorta segments had identical medians (4 [IQR 4, 5]), with means of 4.09 (±0.79 SD) and 4.04 (±0.97 SD), respectively. Aortic arch and descending thoracic aorta segments showed marginally lower means (3.95 ± 1.01 and 3.90 ± 0.98, respectively), though their medians remained stable (4 [IQR 3, 5]). Suprarenal aorta and infrarenal aorta segments exhibited slightly higher means (4.11 ± 0.91 and 4.05 ± 1.04, respectively), both with medians of 4 [IQR 4, 5]. Friedman test analysis confirmed that these differences in scores across segments were not statistically significant (χ²(5) = 7.93, p = 0.16), indicating consistent segmentation performance throughout the aortic anatomy.

### 3.2 Per-category analysis

**Fig. 4:**
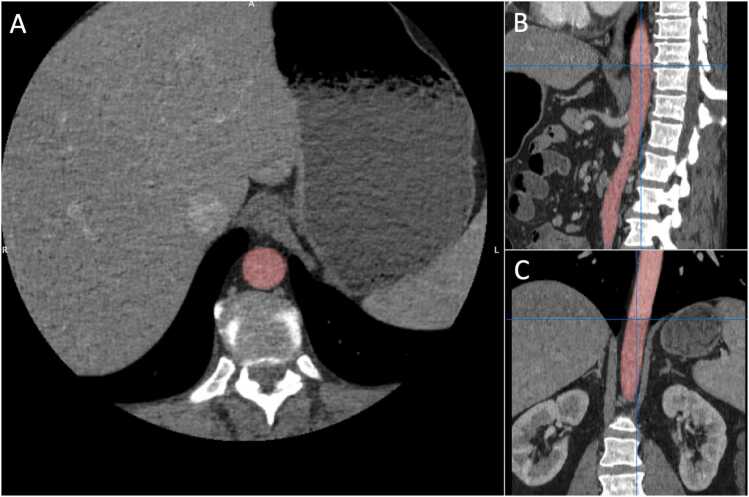
Example of perfect segmentation of the thoracic aorta in the young category. MPR view axial (A), sagittal (B) and coronal (C), mediastinal window with segmentation overlay (red, 80% transparency).

**Fig. 5:**
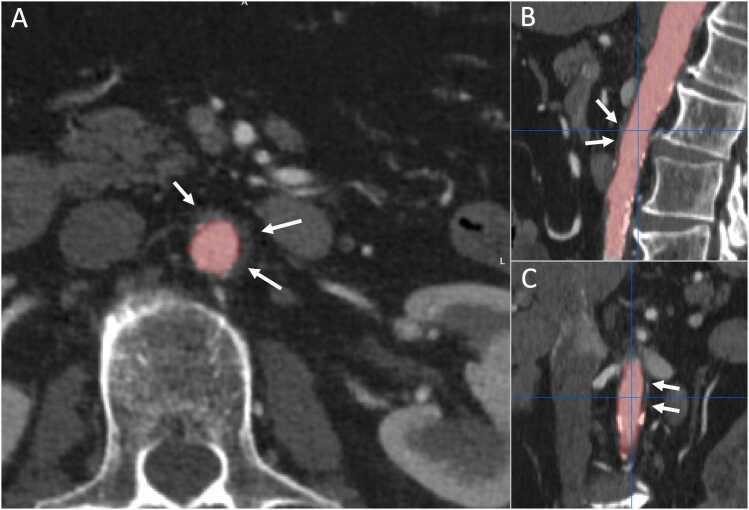
Example of moderate segmentation gaps in the old group, infrarenal abdominal aorta with non-segmented aortic atherosclerotic plaques (white arrows). MPR view axial (A), sagittal (B) and coronal (C), mediastinal window with segmentation overlay (red, 80% transparency).

**Fig. 6:**
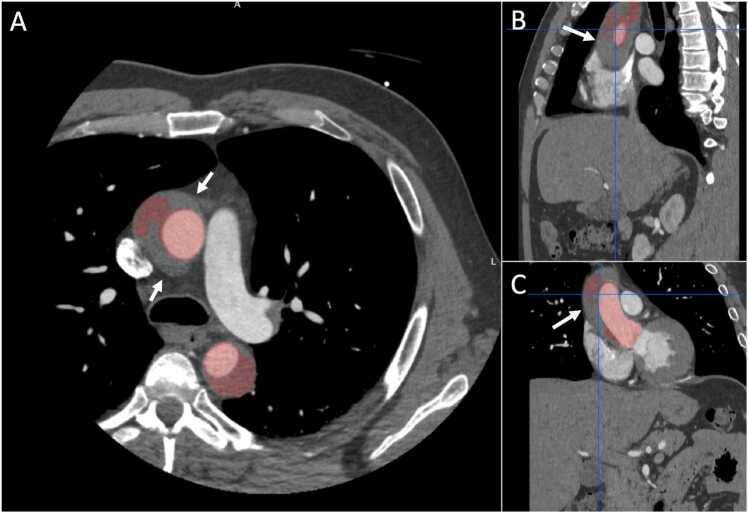
Example of large segmentation errors in a dissection case, showing non-segmented and misclassified aortic regions. MPR view axial (A), sagittal (B), and coronal (C), mediastinal window with segmentation overlay (red, 80% transparency).

**Fig. 7:**
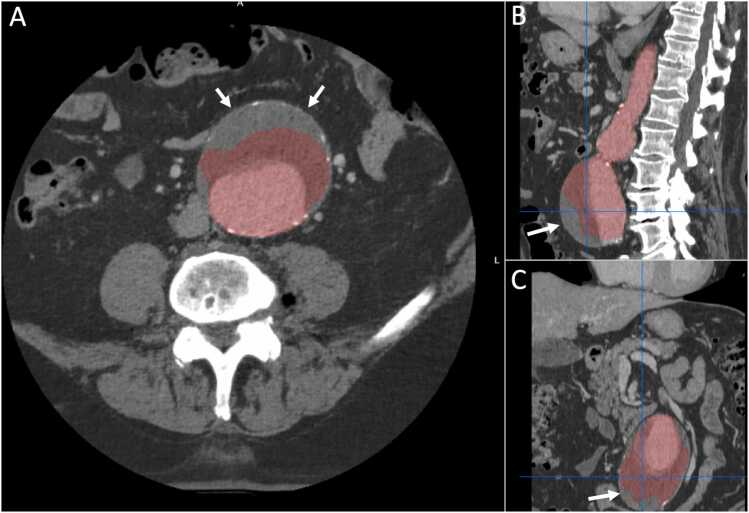
Example of large segmentation errors in an aneurysm case, highlighting regions of under-segmentation (white arrows). MPR view axial (A), sagittal (B), and coronal (C), mediastinal window with segmentation overlay (red, 80% transparency).

Stratification by patient/pathology categories revealed more pronounced differences. Young patients (<30 years) group had the highest median score (5 [IQR 4, 5], mean 4.58 ± 0.53) (Fig. 4)**,** while Old Patients (>70 years) group scored lower (median 4.5 [IQR 4, 5], mean 4.33 ± 0.81) (Fig. 5). Aortic Dissection cases scored the lowest (median 3 [IQR 3, 4], mean 3.22 ± 1.08) (Fig. 6), followed by Aortic Aneurysm (median 4 [IQR 3, 4], mean 3.61 ± 0.94) **(**Fig. 7**)**. The absence of proper arterial enhancement in the aorta did not impair the algorithm’s performance, as venous and non-contrast categories performed well (medians 4 [IQR 4, 5], means 4.18 ± 0.74 and 4.23 ± 0.83, respectively). Friedman test analysis confirmed statistically significant differences in segmentation scores across categories (χ²(5) = 100.77, p < 0.001). Post-hoc Wilcoxon pairwise comparisons with Bonferroni correction revealed that the Dissection group differed significantly from nearly all other groups (p < 0.001), while the Young and Aneurysm groups also showed significant differences with multiple other groups. In contrast, the Old, Venous, and Non-contrast groups exhibited fewer significant differences among themselves.

### 3.3 Quantitative analysis

The quantitative evaluation of segmentation errors revealed a relationship between the qualitative scores and the magnitude of segmentation errors discrepancies. For segments rated as grade 5 (perfect alignment), the mean maximum segmentation error was minimal (0.3 ± 0.6 mm). Segments with grade 4 (minimal error) showed slightly larger discrepancies (3.0 ± 0.9 mm). Moderate errors (grade 3) averaged 6.2 ± 0.9 mm, while grade 2 (large errors) exhibited substantial deviations (15.1 ± 5.0 mm). The most severe failures (grade 1) had a mean maximum error of 29 ± 0 mm, reflecting complete segmentation breakdown in affected regions.

The Spearman’s rank correlation revealed a significant negative relationship between the segmentation scores and the maximal segmentation error (ρ = −1, p = 0.01667). This indicates that as the quality score of the segmentation decreases, the size of the error increases significantly. In particular, lower quality segments (with lower scores) are associated with larger error, suggesting that the precision of the AI's segmentation deteriorates as the quality decreases.

### 3.4 Inter-reader agreement

Inter-observer agreement for qualitative segmentation scores, assessed using weighted Cohen’s kappa, varied across aortic segments and patient categories. Overall, the total agreement across all segments was substantial (κ = 0.72, p < 0.001). Per-segment analysis revealed substantial agreement for the aortic arch (κ = 0.73), descending aorta (κ = 0.70), and suprarenal aorta (κ = 0.80), while agreement was moderate for the ascending aorta (κ = 0.53) and infrarenal aorta (κ = 0.58). The aortic root showed only slight agreement (κ = 0.03, p = 0.9). Per-category analysis demonstrated almost perfect agreement for elderly patients (κ = 1.00) and non-contrast scans (κ = 1.00), substantial agreement for aortic dissections (κ = 0.74), and moderate agreement for aneurysms (κ = 0.48). Fair agreement was observed in young patients (κ = 0.37) and venous-phase scans (κ = 0.23).

## 4 Discussion

In this study, we demonstrated that TotalSegmentator-AI achieved overall adequate performance for aortic segmentation in routine clinical CT imaging, with most aortic segments showing perfect or near-perfect alignment with the true anatomical contours. Identifying where the algorithm fails in pathological scenarios constitutes the primary scientific core of this study. Conversely, our results show that TotalSegmentator-AI performs best in young patients with morphologically normal aortas, a scenario where automated segmentation adds the least clinical value, as these patients rarely require dedicated CTA studies or complex vascular interventions. By highlighting the substantial degradation of performance in dissections and aneurysms (specifically regarding critical tasks such as true/false lumen differentiation, peripheral thrombus delineation, and precise diameter measurements required for EVAR/TEVAR planning) our findings provide a realistic baseline of current AI limitations. This serves as a strong warning to clinicians against uncritical reliance on automated outputs in clinical settings, where generalist models cannot currently substitute for dedicated, pathology-trained pipelines. While previous studies reported high performance and reproducibility of TotalSegmentator for abdominal and pulmonary segmentation, clinical evaluations of such algorithms remain scarce [14], [15]. To our knowledge, this is the first study to specifically assess TotalSegmentator’s performance for aortic segmentation in a clinical setting.

Our results show that segmentation performance was not uniform across all patient groups and anatomical conditions. Our clinical stress-test revealed that automated segmentation errors in complex aortic diseases are structurally systematic. In aortic aneurysms, TotalSegmentator-AI repeatedly failed in regions of mural thrombosis, tracking only the contrast-enhanced lumen while omitting the peripheral thrombus and true outer wall. In aortic dissections, the algorithm was unable to recognize the intimal flap, resulting in either an incomplete segmentation of a single lumen or a chaotic fusion of both channels.

Integrating these failure modes uncritically into clinical decision-making presents severe risks. In acute dissection, for instance, misclassifying the true and false lumens can lead to catastrophic consequences if a fenestration or a stent-graft is inadvertently planned in the wrong channel, or if the compression and obstruction of vital arterial branches, such as the celiac trunk or renal arteries, are completely overlooked by the automated mask. Similarly, in EVAR/TEVAR planning, accurate characterization of landing zones and thrombus burden is mandatory; an automated tool that collapses true and false lumen boundaries cannot be safely used for device sizing without risking graft mispositioning or sizing failures.

From a workflow perspective, these pathological errors are highly visible to radiologists during multiplanar reconstruction reviews using a transparency overlay. However, while easily detectable, manually correcting a complex 3D volume across dozens of axial slices represents a substantial bottleneck that neutralizes the primary promise of AI to reduce cognitive burden and accelerate throughput.

Consequently, generalist tools like TotalSegmentator-AI must not be deployed as standalone systems for vascular disease quantification or pre-operative planning without strict "human-in-the-loop" quality control. While specialized algorithms have been developed for complex tasks like aortic dissections, they often lack the generalizability and multi-organ capabilities of broader models [16], [17], [18], [19]. This highlights the inherent limitation of generalist deep-learning models trained on idealized datasets and underscores the critical need to share open datasets with a wider range of pathological configurations to improve both generalist and specialized pipelines [20], [21].

Contrast quality did not appear to significantly affect segmentation accuracy. This is an interesting finding; indeed, from a radiologist’s perspective, good arterial-phase contrast is generally considered essential for high-quality aortic imaging. One might therefore expect that this parameter would be critical for TotalSegmentator to correctly identify the aorta. However, the algorithm performed well even on non-contrast scans. This finding opens an intriguing potential for AI in the analysis of aortic pathologies without contrast, a task that remains challenging for radiologists but may be achievable with automated algorithms [22], [23], [24].

Future work should focus on several directions. First, incorporating more diverse and pathology datasets including cases with dissections, aneurysms, lower-quality imaging, and the presence of implanted devices such as stent grafts, will be essential to improve algorithm efficiency. Indeed, TotalSegmentator was recently updated to provide enhanced segmentation of cardiovascular structures [25], which may lead to improved results compared to those reported here. Furthermore, the exceptionally low inter-reader agreement observed at the aortic root highlights the intrinsic complexity of this transition zone, where boundaries are often anatomically ambiguous on CT. This high subjectivity and variability between readers demonstrates that generalist multi-organ models remain unreliable at the cardiac-vascular junction, warranting the development of dedicated fine-tuning or specialized, pathology- and cardiac-inclusive segmentation pipelines for this specific portion of the aorta. Second, exploring the influence of imaging parameters, such as the field of view (FOV) or ECG-gated images, may help achieve more precise vascular measurements. Importantly, AI performance has the inherent advantage of being able to improve over time through algorithmic advancements and the increasing availability of datasets, offering the prospect of developing powerful, clinically useful algorithms even for complex cases in the future [26], [27]. Finally, the open-source availability of TotalSegmentator makes it particularly suitable for comparative benchmarking studies [28]. Applying the same dataset to other open-source aortic or vascular segmentation tools would enable objective comparisons of performance generalizability, and failure modes.

This study has several limitations. First, it is a monocentric study with a relatively small sample size, which was balanced across predefined categories rather than reflecting a true clinical distribution. While this small sample size precludes the assessment of subtle subgroup heterogeneity, the systematic and repetitive nature of the segmentation failures observed across all pathological cases successfully fulfills the objective of our clinical stress-test, establishing a clear baseline for current AI limitations. Second, all 60 scans were acquired using a single scanner model from a single vendor with a highly standardized imaging protocol. Although this homogeneity ensures high internal validity and isolates the algorithm's performance from technical noise, it limits the external validity of our findings. TotalSegmentator-AI's robustness against variations in scanner vendors, slice thicknesses, different reconstruction kernels, or non-ECG-gated protocols remains untested in our dataset and warrants future multi-centric validation. Third, we did not generate ground truth segmentations. Instead, we prioritized a reader-centric, qualitative and quantitative clinical grading system to evaluate the tool's actual utility in a real-world workflow. Additionally, the quantitative millimeter measurements were performed by one of the primary qualitative readers on the worst-performing segments; this non-independence between the subjective and objective grading scales likely contributed to the perfect negative correlation observed in our statistical analysis. Finally, the lack of repeated measurements by the same reader prevented the formal assessment of intra-observer variability, although inter-reader agreement was thoroughly evaluated and found to be substantial overall.

## 5 Conclusion

In conclusion, this study demonstrates that TotalSegmentator-AI provides highly accurate aortic segmentation in normal anatomy, particularly in younger patients. However, its performance significantly degrades in complex pathological cases such as dissections or aneurysms, rendering it inadequate for unsupervised clinical use or automated vascular planning. These findings underscore that generalist multi-organ segmentation models cannot substitute for dedicated, pathology-inclusive pipelines, and highlight the critical need for comprehensive human-in-the-loop quality control to ensure patient safety in clinical routine.

## CRediT authorship contribution statement

**Guillaume Fahrni:** Writing – review & editing, Visualization, Validation, Supervision, Resources, Project administration, Methodology, Conceptualization. **Rotzinger David C:** Writing – review & editing, Validation, Resources, Project administration, Methodology, Conceptualization. **Marianna Gulizia:** Writing – review & editing, Visualization, Investigation, Data curation. **Chiara Pozzessere:** Writing – review & editing, Resources, Methodology, Investigation. **Dania El Rahal:** Writing – original draft, Resources, Methodology, Investigation, Formal analysis, Data curation.

## Ethics statement

The Legal Affairs Unit of CHUV has been confirmed that this study falls outside the scope of the Swiss research legislation (Federal Act on Research involving Human Beings, Human Research Act, HRA, SR 810.30) and therefore does not require ethics committee authorization, in accordance with Swiss legislation and institutional guidelines.guidelines.

## Declaration of Competing Interest

The authors declare that they have no known competing financial interests or personal relationships that could have appeared to influence the work reported in this paper.

## Data Availability

The anonymized aortic CTA dataset (AortaSeg-60) generated and analyzed during this study is publicly available on the Zenodo repository: 10.5281/zenodo.18147026.
